# Fully integrated topological electronics

**DOI:** 10.1038/s41598-022-17010-8

**Published:** 2022-08-04

**Authors:** Yuqi Liu, Weidong Cao, Weijian Chen, Hua Wang, Lan Yang, Xuan Zhang

**Affiliations:** 1 Department of Electrical and Systems Engineering, Washington University, St Louis, MO USA; 2 School of Electrical and Computer Engineering, Georgia Institute of Technology, Atlanta, GA USA; 3 Department of Information Technology and Electrical Engineering, Swiss Federal Institute of Technology Zurich, Zurich, Switzerland

**Keywords:** Electrical and electronic engineering, Topological insulators

## Abstract

Topological insulators (TIs) have attracted significant attention in photonics and acoustics due to their unique physical properties and promising applications. Electronics has recently emerged as an exciting arena to study various topological phenomena because of its advantages in building complex topological structures. Here, we explore TIs on an integrated circuit (IC) platform with a standard complementary metal-oxide-semiconductor technology. Based on the Su–Schrieffer–Heeger model, we design a fully integrated topological circuit chain using multiple capacitively-coupled inductor–capacitor resonators. We perform comprehensive post-layout simulations on its physical layout to observe and evaluate the salient topological features. Our results demonstrate the existence of the topological edge state and the remarkable robustness of the edge state against various defects. Our work shows the feasibility and promise of studying TIs with IC technology, paving the way for future explorations of large-scale topological electronics on the scalable IC platform.

## Introduction

Topological insulators (TIs) are a new quantum state of matter where a material behaves as an insulator in the interior yet as a conductor on the boundary^1^. Particularly, the conductive edge state is protected by time-reversal symmetry and is robust to perturbations from surface imperfections or local disorders. These materials were first found in the field of condensed matter physics by studying the quantum Hall Effect^2^. Since then, they have attracted significant attention from the scientific community. In the past decades, TIs have been extensively studied in the classical wave fields, such as photonics^3–15^, acoustics^16–20^, plasmonics^21,22^, and mechanics^23,24^. A number of intriguing effects and applications have been proposed and investigated, including spintronics devices^1^, superconducting proximity effect^25^, infrared detectors and thermoelectric applications^26^, purely electric magnetic memory writing and dissipationless electronics^27^, and topological quantum computing^28^.

**Figure 1:**
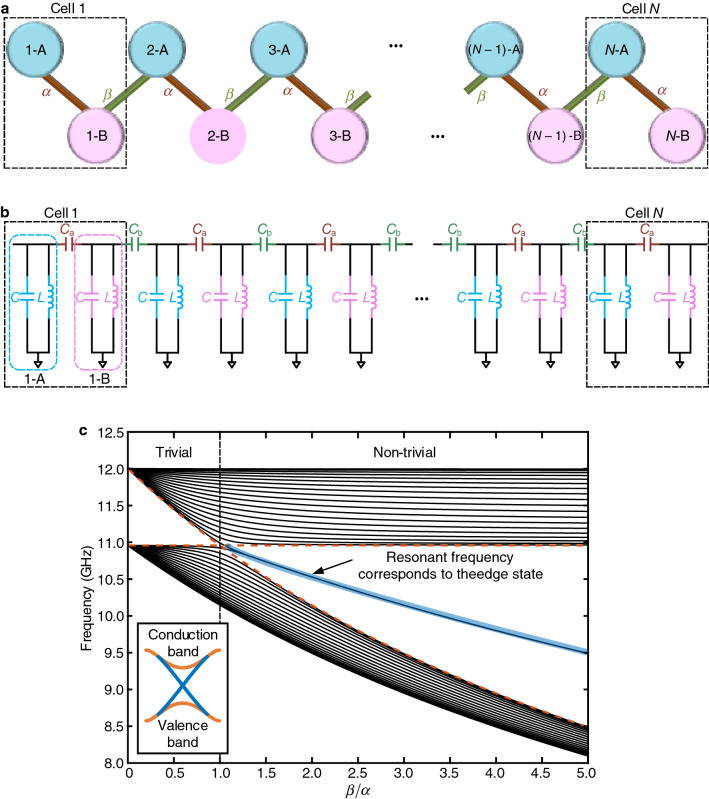
Illustration of the theoretical SSH model and one of its equivalent electronic circuit models. (**a**) A general theoretical 1-D SSH model consisting of *N* cells. Each blue/pink circle represents a sub-unit A/B in the *i*th cell. (**b**) The schematic of an equivalent electronic circuit (i.e., a 1-D circuit chain) for the theoretical SSH model. Each cell in the 1-D circuit chain consists of two LC resonators coupled by a linear capacitor $C_\text {a}$. Each cell is coupled to one another by a linear capacitor $C_\text {b}$. All inductance and capacitance in different LC resonators are the same. **c**, Numerical simulation of the 1-D SSH circuit chain with 20 cells. The characteristic frequency is $f_c=\omega _c/(2\pi )=12$ GHz. $C_\text {a}$ is fixed at the designed value and $C_\text {b}$ is varied from 0 to $5C_\text {a}$. Orange dashes outline the bandgap edge frequencies calculated from $\sqrt{1/(1+2\alpha )}f_c$ and $\sqrt{1/(1+2\beta )}f_c$. The vertical black dash line is the boundary between nontrivial and trivial regimes at $\beta /\alpha =1$. The edge state frequency at $\sqrt{1/(1+\alpha +\beta )}f_c$ is highlighted in blue. The inset is a conceptual diagram of a TI’s energy bands. Orange curves correspond to the bulk bands, and the blue curves correspond to the edge states at the surface.

Electronics^29–49^ has recently emerged as an excellent platform to study TIs due to its advantages in easy probing, reliable fabrication, and flexible tuning of electronic devices. Prior arts^29–49^ have reported the existence of topological edge-state-like behaviors in various electronic circuits, ranging from simple inductor–capacitor ladders^35,40–43^ to complex circuit networks^29,30,32,34,36–39,44–46,48^. Particularly, electronic circuits based on the Su–Schrieffer–Heeger (SSH) model^50^ have been widely used to study TIs. A generic one-dimensional (1-D) SSH chain is shown in Fig. 1a. It consists of *N* cascaded cells, each of which hosts two sub-units (A and B). Intra-cell hopping amplitude $\alpha$ and inter-cell hopping amplitude $\beta$ describe the strength of the bonds within and between cells, respectively. When the inter-cell coupling $\beta$ is stronger than the intra-cell coupling $\alpha$, the chain is topologically nontrivial and possesses an edge state; otherwise, the edge state disappears and only bulk states are present. This edge state, protected by time-reversal symmetry, is immune to various kinds of perturbations and disorders^51^. Such a 1-D model can also be spatially extended to high-dimensional structures, such as two-dimensional (2-D) lattice, three-dimensional (3-D) honeycomb^32^, breathing pyrochlore^29^, graphene^43,52^, and Weyl structures^48^.

So far, the implementations of topological electronic circuits have been limited to printed circuit boards^38–49^ with discrete components. These electronic platforms are constrained by low operating frequencies at a few megahertz as well as excessive parasitics and have difficulty in scaling to small physical dimensions and diverse integrated structures. Integrated circuit (IC) technology as leading nanotechnology for electronics, is capable of covering a wide applied spectra ranging from DC to terahertz due to its scalability in the physical size of integrated devices. In addition, IC technology supports the flexible design and provides a standard manufacturing process for complex 2-D or 3-D structures, making it especially attractive and promising to explore TIs. Here, we study topological electronics on ICs by designing a 1-D SSH circuit chain with a 130-nanometer (nm) complementary metal-oxide-semiconductor (CMOS) process. We show the detailed circuitry design and perform comprehensive post-layout simulations to characterize the topological properties of the chain. Our results clearly demonstrate the topological edge state of the chain and its robustness against various defects. Our study lays a foundation for exploring large-scale topological electronics with the scalable IC platform.

## Results

### Theoretical model

The 1-D SSH circuit chain corresponding to the theoretical SSH model is shown in Fig. 1b. It consists of multiple serially-connected inductor–capacitor (LC) cells. Each cell is composed of a pair of LC resonators with the same inductance *L* and capacitance *C*. Both the intra-cell hopping $\alpha$ and inter-cell hopping $\beta$ are achieved by capacitive coupling. Particularly, the intra-cell coupling of a pair of resonators in a cell is realized by a linear capacitor with capacitance $C_{\text {a}}$ while the inter-coupling between two cells is attained by a linear capacitor with capacitance $C_{\text {b}}$. Fig. 1c shows a numerical characterization of the 1-D SSH circuit chain with twenty cells by fixing *L*, *C*, $C_{\text {a}}$ and changing $C_{\text {b}}$ to vary $\beta$ in the range of $0{-}5C_{\text {a}}$ (i.e., $\beta /\alpha \in (0,5)$). It can be observed that the chain has two bulk bands with multiple bulk frequency modes within the band. In the case when $\beta <\alpha$, there are no edge states in the band gap because the chain is topologically trivial; otherwise, the chain is topologically nontrivial and an edge state appears–a resonant frequency (highlighted in blue in Fig. 1c) emerges between the two bands. This frequency corresponds to the edge state of the SSH model. Such bulk and edge resonant frequencies are analogous to the energy band levels in a TI where conducting edge energy states emerge in the insulator bandgap (Fig. 1c inset). Therefore, the topological properties of the 1-D SSH circuit chain are consistent with the theoretical 1-D SSH model (Methods). Note that a more thorough characterization is attached in Supplementary Information (Supplementary Note 2), where the Chiral symmetry of the circuit is discussed.

### System design

**Figure 2:**
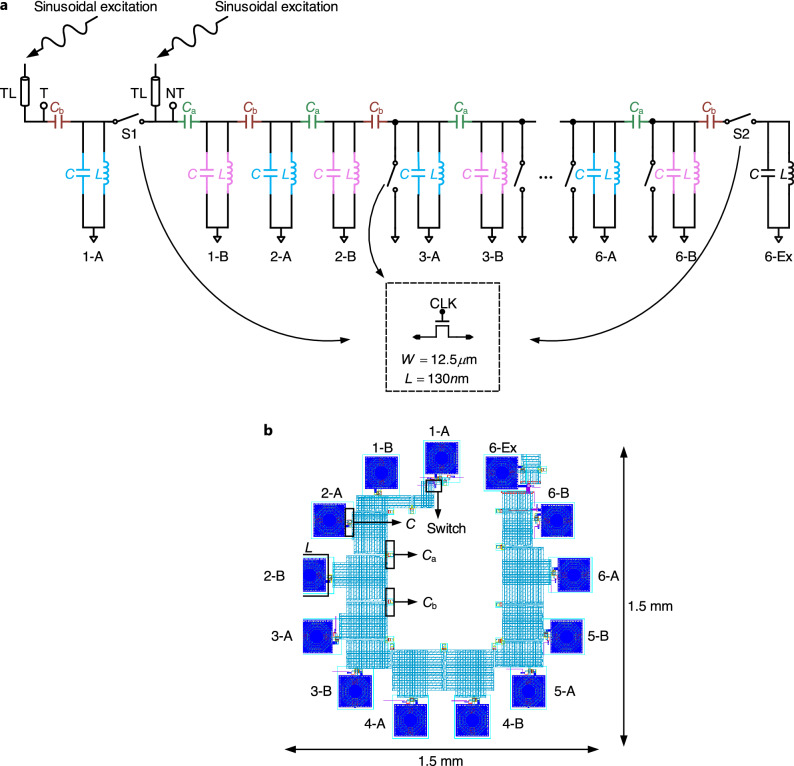
Implementation of the 1-D SSH circuit chain. (**a**) Schematic of the six-cell 1-D SSH circuit chain. In each LC resonator, *L* = 1 nH and *C* = 163 fF. Intra-cell coupling capacitance is $C_\text {a}={34}$ fF and inter-cell coupling capacitance is $C_\text {b}={16}$ fF. The trivial and nontrivial setups are probed at the points T and NT, respectively. Sinusoidal signals are sourced into the chain via the transmission lines (TLs). The inset shows the schematic of the on-chip switch. (**b**) Core physical layout of the six-cell 1-D SSH circuit chain with labeled dimensions and exemplary components (i.e., capacitor, inductor, and switch).

For simplicity, we use six LC cells^42^ to build our topological circuit chain as a proof-of-concept implementation. However, it should be noted that the number of LC cells can be readily scaled to more than six on the IC platform. All capacitors used in the chain are single-plate nitride metal–insulator–metal capacitors (mimcap). All inductors are single-layer symmetric inductors (symind). According to Fig. 1c, the edge state only emerges in the nontrivial regime when $\beta >\alpha$, i.e., the capacitance value $C_{\text {b}}$ is greater than $C_{\text {a}}$. In order to contrast with the trivial regime without the presence of the edge state, we incorporated on-chip switches into the design to flexibly transform between the trivial and nontrivial structure of the chain. Fig. 2a shows the detailed circuit schematic. We added an extra sub-unit 6-Ex to the end of the chain and placed a switch S1 between the sub-unit 1-A and 1-B, as well as another switch S2 between 6-B and 6-Ex. By disconnecting the first sub-unit (1-A) from the chain (turning off S1) and connecting the extra sub-unit (6-Ex) at the end (turning on S2), the inter-cell coupling and intra-cell coupling are essentially swapped. These operations give rise to a chain that can operate in a nontrivial regime. In this case, 1-B and 6-Ex play the role of 1-A and 6-B, respectively, in the trivial chain.

To test the robustness of our chain, we placed extra switches between each sub-unit from 3-A to 6-A and the ground to flexibly introduce short-circuits (i.e., defects)^40,42,46^ on demand. The switch schematic is shown in the subset of Fig. 2a. These switches are delicately designed with standard transistors in order to minimize parasitics. One single NMOS switch is sufficient to pass the small sinusoidal signals intended in our system characterization. The core physical layout of our 1-D SSH circuit chain is shown in Fig. 2b. It occupies an area of 1.5 mm $\times$ 1.5 mm in a 130 nm CMOS process. Wide metal traces on the thick aluminum metal layer are used for routing to reduce unwanted parasitic resistance of the physical layout. All results reported below except those in Fig. 4b–d are obtained from performing post-layout simulations on the physical layout of the 1-D SSH circuit chain.

IC physical layout (also known as IC mask layout, or mask design) is the representation of an IC in terms of planar geometric shapes corresponding to the different stacked physical layers (e.g., metal, oxide, or semiconductor) during the fabrication process. A semiconductor foundry uses this physical layout information to generate the photomasks required by the photo-lithographic process for chip fabrication. The post-layout simulations can accurately extract the precise parasitics from an IC physical layout and are therefore considered a golden standard to verify its function. For analog IC designs (e.g., our 1-D SSH circuit chain) which are sensitive to parasitic effects, the post-layout simulation results from a high-fidelity simulator often match well with the measured results from a fabricated chip. In our work, we use Cadence Spectre, an industry-standard design tool for IC design, and foundry provided process design kit (PDK) which contains accurate device and parasitics models, to ensure reliable simulation results.

### Demonstration of topological edge state

**Figure 3:**
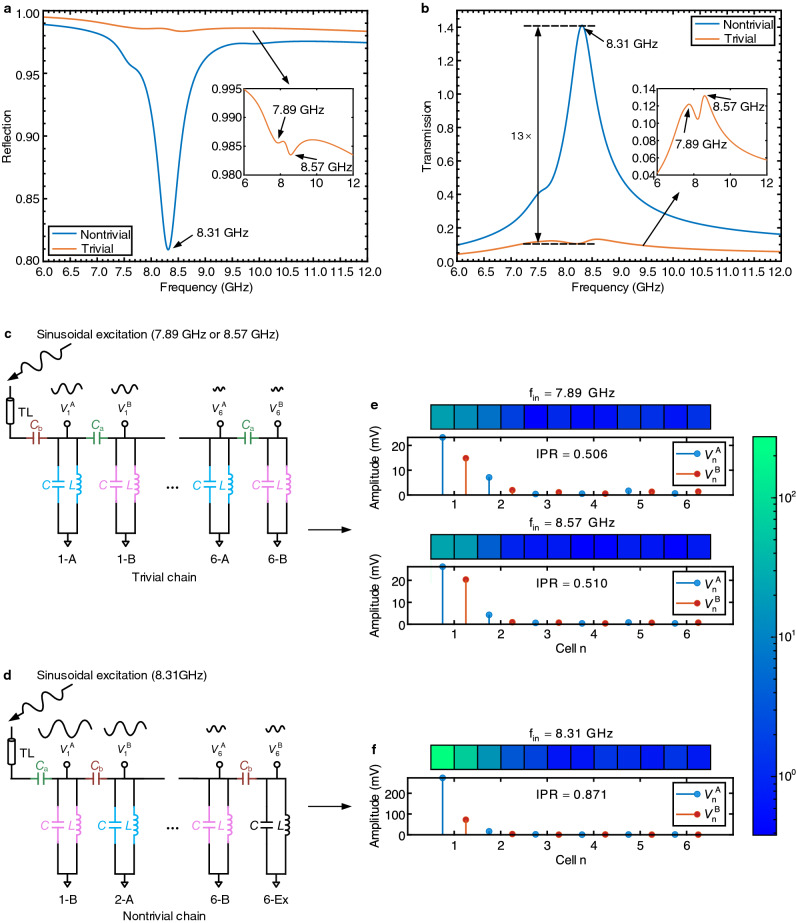
Demonstration of the existence of the edge state. (**a**) Reflection spectra of the nontrivial and trivial chain. (**b**) Transmission spectra of the nontrivial and trivial setup. In (**a**) and (**b**) the insets zoom in the result of the trivial setup. Arrows indicate the frequency location of the edge state and bulk state frequencies. (**c**, **d**) Trivial and nontrivial setup for the simulation of voltage profiles. (**e**, **f**) Voltage profiles of each sub-unit in the 1-D SSH circuit chain under a sinusoidal excitation with a 200 mV amplitude at two bulk state frequencies for the trivial setup and at the edge state frequency for the nontrivial setup. The color map inset shows the voltage amplitude across all sub-units in a log scale. Based on these voltage profiles, the IPR, a quantitative measurement of the degree of localization for each simulation is also calculated and labeled in each sub-figure. A larger IPR of the nontrivial chain shows a more localized profile.

To thoroughly show the existence of the topological edge state, we performed multiple simulations to study the reflection and transmission properties of the chain^40–43,45,46^. First, we simulated the reflection spectrum of the chain, which directly relates to the chain’s input impedance (Eq. 8 in Methods). The resonance at the edge state can significantly alter the input impedance of the chain^42,43,45^. Therefore, obtaining the reflection spectrum can characterize the existence of the edge state. To attain the reflection spectrum, we treated the chain as a one-port network. A transmission line (TL) with a characteristic impedance of $50 \Omega$ was attached to the port. The port locations are labeled as “T” and “NT” in Fig. 2a for the trivial and the nontrivial setup, respectively. We then sourced a frequency-varying sinusoidal signal into the chain via the TL and simulated the reflection spectrum at the port. Fig. 3a shows the reflection spectra of the chain for the two different set-ups. For the trivial set-up, the reflection spectrum exhibits slight dips at two bulk state frequencies (7.89 GHz and 8.57 GHz). However, for the nontrivial set-up, the reflection spectrum shows only one sharp dip at the edge state frequency (8.31 GHz) that does not appear in the trivial counterpart. The comparison suggests that the impedance at the edge state frequency is remarkably different from the one at the bulk states, confirming the existence of the topological edge state. Note that due to the nonidealities (e.g., parasitics) of devices (Supplementary Note 2) and the slight difference between bulk mode frequencies, most bulk state frequencies are overlapped and we mainly observed two bulk state frequencies during the simulations.

Second, we simulated the transmission spectrum of the chain. During the simulation, we sourced a frequency-variable sinusoidal signal into the chain via the TL and monitored the voltage magnitude of the transmitted wave at the edge site, i.e., 1-A (1-B) for the trivial (nontrivial) chain in Fig. 2a. Fig. 3b shows the transmission spectra (Eq. 9 in Methods) of the chain for the two set-ups. The magnitude at the edge state frequency (8.31 GHz) of the nontrivial chain shows a high peak; while the magnitudes at the bulk state frequencies (7.89 GHz and 8.57 GHz) of the trivial chain exhibit multiple slight peaks. The ratio between these peaks is as large as 13, and can be attributed to the localized wave function at the edge site in the SSH model^53^.

To further verify the localized wave function of the edge state, we simulated the voltage amplitude of each sub-unit (i.e., each LC resonator in a cell) in the chain. The voltage amplitude distribution of all sub-units under a sinusoidal excitation at the edge or bulk state frequency reflects the wave function at that particular state^40–43,46^. To obtain such an amplitude distribution, we adopted a method similar to the previous reflection/transmission spectrum simulation. Instead of using a frequency-varying sinusoidal source, we applied an amplitude-varying sinusoidal source with a fixed frequency to this simulation. We sourced the amplitude-varying sinusoidal signal into the chain via the TL and monitored the wave amplitude at each sub-unit. At the trivial setup (Fig. 3c), the chain was excited at the two bulk state frequencies (7.89 GHz and 8.57 GHz); whereas at the nontrivial setup (Fig. 3d), the chain was excited at the edge state frequency (8.31 GHz). Simulated voltage distributions of both the trivial and the nontrivial setup under sinusoidal inputs with a 200 mV amplitude are shown in Fig. 3e, f. Based on these voltage distributions, the inverse participation ratio (IPR, a quantitative measurement of the degree of localization, see Eq. (6) in Methods) for the edge or bulk state frequency is also calculated and labeled in Fig. 3e, f. A larger IPR indicates a more localized profile. Compared to the trivial chain, the voltage distribution of the nontrivial chain is strongly localized at the edge unit and decays rapidly into the bulk units, verifying the presence of the edge state.

### Robustness of the topological edge state

**Figure 4:**
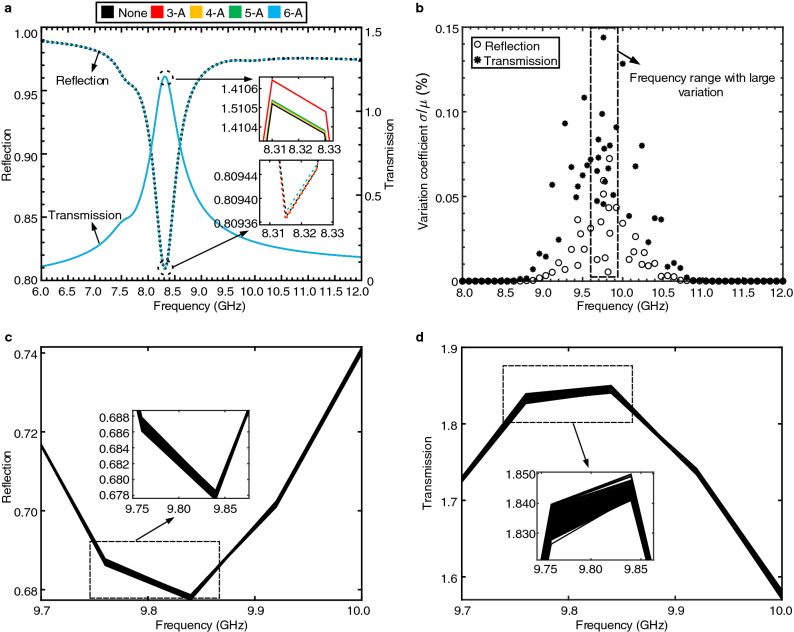
Demonstration of the robustness of the edge state. (**a**) The reflection and transmission spectra of the nontrivial chain when different sub-units are short-connected to the ground. The top/bottom inset shows the zoomed-in view of the transmission/reflection spectra of the circle region that covers the neighbor of the edge state frequency. The tiny variations of the reflection and transmission spectra across different short-connected positions indicate that the presence of defects almost does not affect the behavior of the chain. (**b**) The variation coefficients obtained from 500 rounds MC simulation for each frequency point in a sweeping range of 8.0–12.0 GHz that covers the edge state frequency. The asterisk and the circle indicate the reflection and transmission, respectively. The rectangular box here shows the frequency range where the variation coefficients are significantly larger than that of other frequency ranges. (**c**, **d**) The reflection and transmission spectra of 500 rounds of MC simulation in the frequency range highlighted by the rectangular box in (**b**). These spectra almost do not differ from each other, indicating the ignorable effect from the random device variations resulted by the practical fabrication process.

To demonstrate the robustness of the edge state, two kinds of defects were used. First, sub-units were short-connected to the ground to intentionally introduce defects into the chain^40,42,46^. In the simulation, we alternately short-connected sub-unit 3-A, 4-A, 5-A, and 6-A to the ground by turning on the corresponding switch as shown in Fig. 2a. Simulations were then performed on the nontrivial chain to obtain the reflection and transmission spectra at its input port. The edge state was clearly observed as significant depths or peaks across different defective locations (Fig. 4a). Moreover, even with the presence of these defects, the edge state still existed with ignorable frequency change, exhibiting strong robustness.

Second, IC inherently suffers from imperfect manufacturing processes, giving rise to the random variations of fabricated devices. Such random device variations introduce disorders into our SSH circuit chain. They are treated as the second kind of defect imposed on the components in our chain. To study the effect of such a defect on the edge state, we performed Monte-Carlo (MC) simulation on the nontrivial circuit chain. Analogous to random variations introduced in hopping amplitudes^51^, we applied variations to all components within and connected to units 3–6. MC simulation is a circuit-level simulation that can mimic practical fabrication variations by randomly sampling device parameters from their statistical distributions. In our study, 500 rounds of MC simulation were used to obtain the reflection and transmission spectra of the nontrivial chain around the edge state frequency. At each frequency point, we calculated the standard deviation $\sigma$ and mean $\mu$ for both the reflection and transmission magnitude based on the 500 rounds of MC simulation. We then defined a variation coefficient as the ratio between the standard deviation $\sigma$ and mean $\mu$ (Eq. 7 in Methods) to represent the robustness of the edge stage frequency against the random device variations. Note that for the MC simulations here, we adopt pre-layout simulation (without considering parasitics) for fast characterizations. Each round of post-layout simulation (considering parasitics) takes tens of minutes, making it time-consuming to obtain results of 500 rounds of MC simulation.

Fig. 4b shows the variation coefficient at each frequency point in the sweeping range from 8.0 to 12.0 GHz that covers the edge state frequency. Note that without considering parasitics, the edge state frequency shifts to a higher frequency, i.e., 9.84 GHz. For all rounds of MC simulation, the maximum variation coefficient for the reflection spectra magnitude is 0.05% and 0.15% for the transmission spectra magnitude. The rectangular box in Fig. 4b indicates the frequency range where the variation coefficients are significantly larger than that of other frequency ranges. Fig. 4c, d highlight the reflection and transmission spectra of the 500 rounds of MC simulation in the frequency range labeled by the rectangular box. The results suggest that these spectra almost overlap with each other. Their variations cause negligible effect. Such small variation coefficients show that the edge state frequency almost remains unchanged even with the presence of random device variations. Conventionally, the manufacturing variations could often lead to the deviation of a fabricated device value from its nominal value by ± 10%^54^, giving rise to significant performance degeneration of circuits. For example, the variation coefficient for the frequency response of a resonator built upon the LC sub-unit of our chain could be up to 10.2% due to the device variations^55^. And there are limited ways to overcome these downsides brought by the manufacturing variations. Our study shows that topological protection could provide a new avenue to effectively conquer these limitations by maintaining the resonant frequency. Further results we have obtained again confirm the robustness of the topological edge state frequency to temperature variations (Supplementary Note 5).

## Conclusion

We have reported a 1-D SSH circuit chain based on a 130 nm CMOS technology. Multiple high-fidelity post-layout simulations have been performed to study the topological properties of the chain. We first show the existence of the edge state from different aspects, i.e., scattering coefficients (refection and transmission spectra) and transient behaviors (voltage amplitude distribution). We then demonstrate the robustness of the topological edge state by introducing different defects into the chain. Particularly, we show that the topological edge state is robust against the inherent manufacturing fabrication variations. This finding could open a new way to maintain IC performance resulted by the imperfect fabrication process which still remains a challenge for conventional methodologies to tackle. Given the scalability of IC technology in both physical size and spatial structure, the simple 1-D chain could be readily extended to more complex structures with more advanced technology, e.g., 2-D array and 3-D lattice^29,38,43,44^, to further reveal the interesting more advanced topological phenomena in a higher-frequency domain. Our work shows the promise of applying the scalable IC platform to study TIs, paving ways to enable more explorations such as non-Hermiticity topological electronics^56,57^ and large-scale active topological electronics as well as a number of novel on-chip applications, such as topological wave generation.

## Methods

### Theoretical Su–Schrieffer–Heeger model and the corresponding circuit chain

The Hamiltonian of the theoretical SSH model is expressed as a matrix form as below

1 $$\begin{aligned} {\mathscr {H}}= \left[\begin{array}{llllll} 0 &{} \alpha &{} 0 &{} 0 &{} \dots \\ \alpha &{} 0 &{} \beta &{} 0 &{} \dots \\ 0 &{} \beta &{} 0 &{} \alpha &{} \dots \\ 0 &{} 0 &{} \alpha &{} 0 &{} \dots \\ \vdots &{} \vdots &{} \vdots &{} \vdots &{} \ddots \end{array}\right]. \end{aligned}$$

By grouping the wave functions $\psi _i$ on all sub-unit *i* as a vector, the following matrix equation characterizes this chain, where *E* is the eigenenergy.

2 $$\begin{aligned} {\mathscr {H}}\left(\begin{array}{l} \psi _1\\ \psi _2\\ \psi _3\\ \psi _4\\ \vdots \end{array}\right) =E\left(\begin{array}{l} \psi _1\\ \psi _2\\ \psi _3\\ \psi _4\\ \vdots \end{array}\right). \end{aligned}$$

The 1-D SSH circuit chain has the following parameters: two alternating coupling capacitances $C_{\text {a}}$ and $C_{\text {b}}$; inductance *L* and capacitance *C* in each LC resonator. We denote the characteristic angular frequency of each resonator as $\omega _c = 1/\sqrt{LC}$ and the voltage amplitude on *i*th sub-unit A(B) as $V_i^{\text {A}}$ ($V_i^{\text {B}}$). Given that $C_{\text {b}}$ is the inter-cell and $C_{\text {a}}$ is the intra-cell coupling capacitance, we can define the corresponding hopping amplitude $\beta$ ($\alpha$) as the ratio between the coupling capacitance $C_{\text {b}}$ ($C_{\text {a}}$) and the resonator capacitance *C*: $\beta =C_{\text {b}}/C$ ($\alpha =C_{\text {a}}/C$). By applying Kirchoff’s Law to the chain (Supplementary Note 1), the SSH chain preserves angular frequency modes $\omega$ (i.e., angular frequency of the topological trivial or nontrivial modes) governed by the following matrix equation

3 $$\begin{aligned} \left[ {\mathscr {H}}-\left( \alpha +\beta \right) \mathrm {I}\right] \left(\begin{array}{l} V_1^{\text {A}}\\ V_1^{\text {B}}\\ V_2^{\text {A}}\\ V_2^{\text {B}}\\ \vdots \end{array}\right) =\left( 1-\frac{\omega _c^2}{\omega ^2}\right) \left(\begin{array}{l} V_1^{\text {A}}\\ V_1^{\text {B}}\\ V_2^{\text {A}}\\ V_2^{\text {B}}\\ \vdots \end{array}\right). \end{aligned}$$

where I is the identity matrix. If $\beta > \alpha$, the chain is in the nontrivial regime and the edge state emerges in the band gap^53^. The frequency of the edge state can be characterized by the following equation:

4 $$\begin{aligned} \omega _{es}=\sqrt{\frac{1}{1+\alpha +\beta }}\omega _c .\end{aligned}$$

And the edges of the bulk frequency bands are obtained as

5 $$\begin{aligned} \left\{ \sqrt{\frac{1}{1+2\alpha }}\omega _c,\sqrt{\frac{1}{1+2\beta }}\omega _c\right\} \end{aligned}.$$

### Frequency mode calculation

The frequency modes are numerically calculated from the matrix equation Eq. (3) using MATLAB.

### Inverse participation ratio calculation

The inverse participation ratio (IPR) is calculated based on the following formula:

6 $$\begin{aligned} \text {IPR} = \frac{\sum _{i=1}^{N} \left[ \left( V_{i}^{\text {A}}\right) ^4+\left( V_i^{\text {B}}\right) ^4 \right] }{\left( \sum _{i=1}^{N} \left[ \left( V_{i}^{\text {A}}\right) ^2+\left( V_i^{\text {B}}\right) ^2 \right] \right) ^2}. \end{aligned}$$

### Variation coefficient calculation

The variation coefficient *vc*, is defined to be the ratio between the standard deviation $\sigma$ and mean $\mu$:

7 $$\begin{aligned} \text {vc}=\frac{\sigma }{\mu }\cdot 100\%. \end{aligned}$$

### Circuit simulations

The circuit is designed with GlobalFoundries 130 nm CMOS technology library (See Supplementary Table S1 and S2 for more details on component parameters). All simulation results in Figs. 1, 2, 3 and 4 are obtained from the Cadence design suite which is an industry-standard design tool for IC design. Three simulations manners, *ac*, *tran*, and *sp* in Cadence Spectre are used. Particularly, transmission spectra are obtained from *ac* simulation, which is a time-harmonic analysis where the voltage amplitudes on the probed nodes under a sinusoidal input are returned. Transient simulation (*tran* analysis) records the 1-D SSH circuit chain’s response over time, where the voltage waveforms on each sub-unit node under a sinusoidal input are obtained. The MC simulation is performed with *ac* and *sp* simulation. *sp* simulation returns the reflection coefficient defined as below

8 $$\begin{aligned} \Gamma =\frac{Z_L-Z_0}{Z_L+Z_0}. \end{aligned}$$

where $Z_L$ is the impedance of the chain looking into the input port; $Z_0$ is the impedance of the transmission line connected to the chain, which value is set to be 50 $\Omega$. *ac* simulation returns the voltage transmission coefficient between probed nodes and the input source, which is defined as

9 $$\begin{aligned} T=\frac{V_i^{\text {A/B}}}{V_{in}}. \end{aligned}$$

## Supplementary Information

Supplementary Information.
