# Supplementary material for: Fully integrated topological electronics
Source: Sci Rep. 2022 Aug 4;12:13410. doi: 10.1038/s41598-022-17010-8 (PMC9352793; doi:10.1038/s41598-022-17010-8)
Supplement: Supplementary file 1 — Supplementary Information. [file 41598_2022_17010_MOESM1_ESM.pdf]

Supplementary Information

Fully Integrated Topological Electronics

Yuqi Liu, Weidong Cao, Weijian Chen, Hua Wang, Lan Yang and Xuan Zhang

Supplementary Note 1 – Derivation of the circuit mode equation

We take one cell in the 1-D SSH chain shown below as an example to derive the circuit mode equation. By denoting the current flowing through the inductor in sub-unit A and B as I_A and I_B respectively, Kirchhoff's current law can be applied to the two sub-units shown in Supplementary Figure S1. Then, two sets of nodal equations for sub-unit A and B can be obtained.

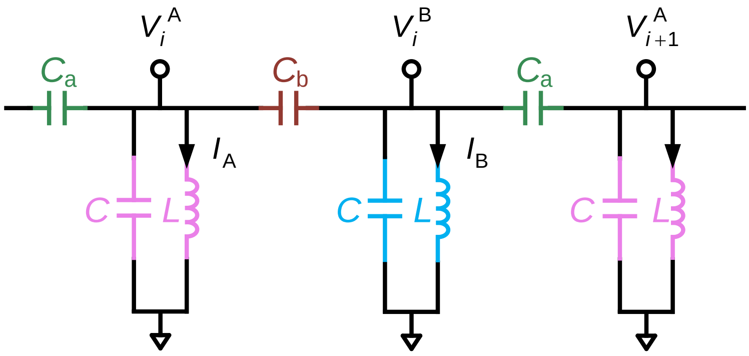

Supplementary Figure S1. Part of the schematic of the1-D SSH circuit chain showing a unit cell and the adjacent sub-unit.

Nodal equations for sub-unit A:

$$L\frac{dI_A}{dt} = V_i^A \tag{1}$$

$$C_A\frac{d(V_i^A - V_{i-1}^B)}{dt} + I_A + C\frac{dV_i^A}{dt} + C_B\frac{d(V_i^A - V_i^B)}{dt} = 0 \tag{2}$$

Nodal equations for sub-unit B:

$$L\frac{dI_B}{dt} = V_i^B \tag{3}$$

$$C_A\frac{d(V_i^B - V_{i+1}^A)}{dt} + I_B + C\frac{dV_i^B}{dt} + C_B\frac{d(V_i^B - V_i^A)}{dt} = 0 \tag{4}$$

Assuming the frequency mode to be in the form of $e^{j\omega t}$, solving (1) and (2) gives $I_A = V_i^A/j\omega L$ and $I_B = V_i^B/j\omega L$. Substituting I_A and I_B in Eq. (2) and Eq. (4) with previously derived formats and rearranging coefficients give the following set of equations for a unit cell in the bulk of the chain:

$$\begin{cases} -(w+v)V_i^A + wV_{i-1}^B + vV_i^B = \left(1-\frac{\omega_c^2}{\omega^2}\right)V_i^A & (5) \\ -(w+v)V_i^B + wV_{i+1}^A + vV_i^A = \left(1-\frac{\omega_c^2}{\omega^2}\right)V_i^B & (6) \end{cases}$$

where $v = C_b/C$, $w = C_a/C$, and $\omega_c = 1/\sqrt{LC}$.

As the edges of the chain are grounded, $V_{i-1}^B = 0$ for the left end and $V_{i+1}^A = 0$ for the right end. The equations for the edge sub-unit 1-A and N-B are shown below:

$$\begin{cases} -(w+v)V_1^A + vV_1^B = \left(1-\frac{\omega_c^2}{\omega^2}\right)V_1^A & (7) \\ -(w+v)V_N^B + vV_N^A = \left(1-\frac{\omega_c^2}{\omega^2}\right)V_N^B & (8) \end{cases}$$

Combining Eq. (7), Eq. (8), Eq. (5), and Eq. (6) for multiple cells arrives at the matrix equation below:

$$\begin{pmatrix} -(w+v) & v & 0 & 0 & \cdots \\ v & -(w+v) & w & 0 & \cdots \\ 0 & w & -(w+v) & v & \cdots \\ 0 & 0 & v & -(w+v) & \cdots \\ \vdots & \vdots & \vdots & \vdots & \ddots \end{pmatrix} \begin{pmatrix} V_1^A \\ V_1^B \\ V_2^A \\ V_2^B \\ \vdots \end{pmatrix} = \left(1-\frac{\omega_c^2}{\omega^2}\right) \begin{pmatrix} V_1^A \\ V_1^B \\ V_2^A \\ V_2^B \\ \vdots \end{pmatrix} \quad (9)$$

This is equivalent to the circuit mode equation in the main text:

$$[\mathcal{H} - (w+v)\mathbb{I}] \begin{pmatrix} V_1^A \\ V_1^B \\ V_2^A \\ V_2^B \\ \vdots \end{pmatrix} = \left(1-\frac{\omega_c^2}{\omega^2}\right) \begin{pmatrix} V_1^A \\ V_1^B \\ V_2^A \\ V_2^B \\ \vdots \end{pmatrix} \quad (10)$$

where the matrix $\mathcal{H}$ is the SSH Hamiltonian:

$$\mathcal{H} = \begin{pmatrix} 0 & v & 0 & 0 & \dots \\ v & 0 & w & 0 & \dots \\ 0 & w & 0 & v & \dots \\ 0 & 0 & v & 0 & \dots \\ \vdots & \vdots & \vdots & \vdots & \ddots \end{pmatrix} \tag{11}$$

The edge states have zero energy which implies

$$\mathcal{H} \begin{pmatrix} V_1^A \\ V_1^B \\ V_2^A \\ V_2^B \\ \vdots \end{pmatrix} = 0 \tag{12}$$

Plugging Eq. (12) into Eq. (10) and solving the equation give the edge state at frequency

$$\omega_{es} = \sqrt{\frac{1}{1 + v + w}}\,\omega_c \tag{13}$$

Supplementary Note 2 – Eigenvalue plot of 1-D SSH circuit chain

For the numerical simulation of the 1-D SSH chain in Figure 1, the eigenvalues derived from the previous section $\left(1 - \frac{\omega_c^2}{\omega^2}\right)$ is shown here as an alternative way to characterize the system by showing the Chiral symmetry.

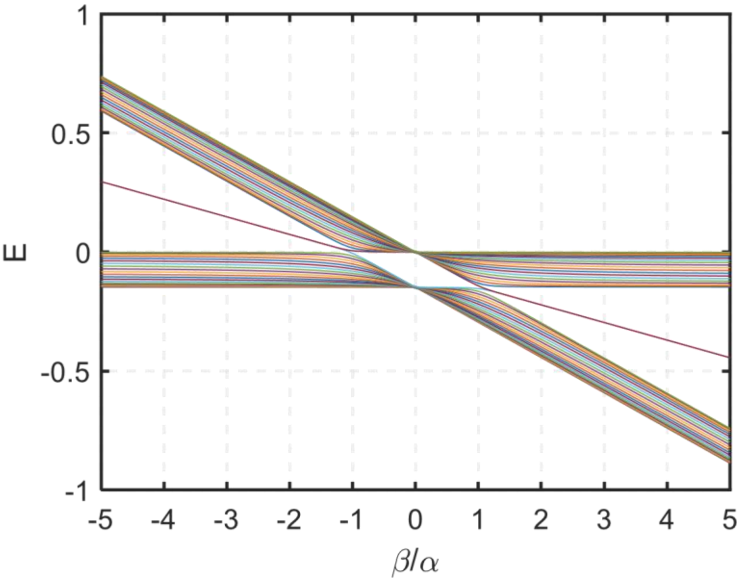

Supplementary Figure S2. Eigenvalue vs capacitance ratio

Negative capacitance ratio is also incorporated here to show the Chiral symmetry. However, it should be noted that the negative capacitance ratio is not physically feasible.

Supplementary Note 3 – Parasitic investigation of inductor *symind*

A realistic inductor contains parasitic elements that can be modeled by an ideal inductor in series with the DC resistance of the inductor and in parallel with another resistance modeling the magnetic loss and a parasitic capacitor, as shown in Supplementary Figure S3.

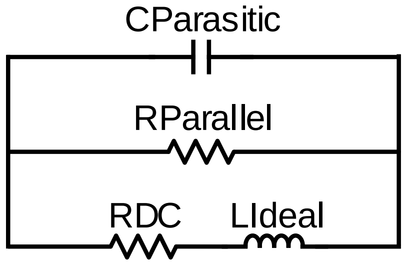

Supplementary Figure S3. Circuit model of a realistic inductor.

For the practical 1-D SSH circuit chain, the parasitic capacitance of the inductor adds into the capacitor of each LC resonator. Therefore, the total capacitance in the resonator increases, resulting in a lower characteristic frequency ω_c than the one predicted by the theoretical model. Correspondingly, the edge state frequency will decrease since it is its proportional to ω_c. This explains the deviation of the realistic chain's edge state frequency from the theoretical value.

The parasitic capacitance CParasitic can be calculated from the measured self-resonant frequency of the inductor f_0 by the following formula:

$$CParasitic = \frac{1}{(2\pi f_0)^2 L} \quad (14)$$

By using the above formula and the measured self-resonant frequency in simulation, the parasitic capacitance of the inductor in the SSH chain turns out to be around 19.83 fF. Supplementary Figure S4 shows the recalculated circuit frequency modes for the six-unit SSH chain after incorporating this parasitic capacitance.

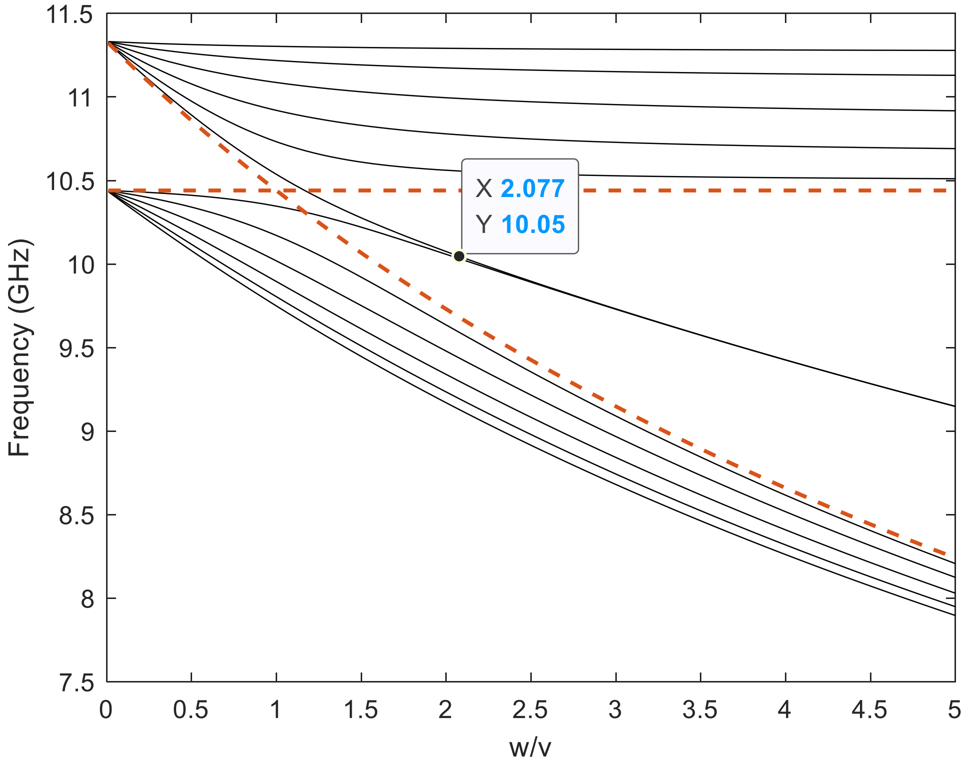

Supplementary Figure S4. Re-calculated eigenfrequencies of the chain with $N = 6$ cells. All the configurations are the same as Figure 1b in the main text except for the number of units and the incorporation of inductor parasitic. The marker shows the approximate edge state frequency for the designed values of C_a and C_b.

After taking the parasitic capacitance of the inductor into consideration, the edge state frequency drops by 0.5GHz from the purely ideal theoretical value. The edge state frequency predicted by pre-layout simulation turns out to be lower by an additional 0.2GHz, due to the non-ideality of capacitor. Therefore, it can be concluded that the parasitic of the inductor affects the realistic circuit chain more than that of the capacitor.

Supplementary Note 4 – Detailed component parameters

The detailed component parameters such as dimensions of the capacitors and inductor used in the circuit are summarized in Supplementary Table S1 and S2 respectively.

Supplementary Table S1. Detailed component parameters of capacitors in the circuit.

Component Name	C_a*	C_b*	C
Library cell name	*mim*		
Capacitance (fF)	67.154	32.536	162.809
Length (μm)	8	5.5	12.5
Width (μm)	8.14	5.66	12.77
Multiplicity	1		
Sub Resistance (Ω)	50		

In the actual circuit designed, C_a and C_b are realized by two capacitors in series to facilitate layout. Therefore, the actual capacitors used C_a* and C_b* are twice the designed values C_a and C_b.

Supplementary Table S2. Component parameters of the inductor in the circuit.

Component Name	L
Library cell name	*symind*
Inductance (nH)	1.08
Outer radius (μm)	150
Spiral width (μm)	10
Space between spiral (μm)	5
# of turns	3
Underpass width (μm)	15

Supplementary Note 5 – Simulation results across temperature

Figure S5 shows the reflection spectra for 500 Monte Carlo sampling runs at three different temperatures: −20 ℃, 27 ℃, and 90 ℃.

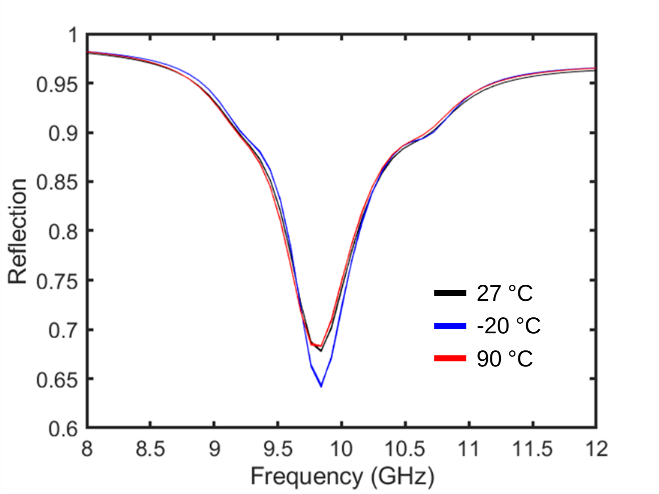

Supplementary Figure S5. Reflection spectra from 500 rounds of Monte-Carlo simulation runs at different temperatures.

The topological edge state still exists, and the edge state frequency is unchanged under temperature changes. However, the input impedance shifts which results in difference in reflection magnitude. This is because inductors will display different quality factors at different temperatures.
